# 2 Differential Transcriptomic Responses Among Burn Injury Outcomes Related to Morality and Hospital Length of Stay

**DOI:** 10.1093/jbcr/irae036.002

**Published:** 2024-04-17

**Authors:** Burook Misganaw, Benjamin Levi, Desiree Pinto, Tuan D Le, Anthony Pusateri, Arti Gautam, Lauren T Moffatt, Jeffrey W Shupp, Rasha Hammamieh

**Affiliations:** Walter Reed Army Institute of Research, Silver Spring, Maryland; University of Michigan, Ann Arbor, Michigan; MedStar Washington Hospital Center, Washington, DC; MedStar Washington Hospital Center, Grand Prairie, Texas; Naval Medical Research Unit San Antonio, JBSA-Fort Sam Houston, San Antonio, Texas; Firefighters’ Burn and Surgical Research Laboratory, Washington, DC; MedStar Health, Georgetown University School of Medicine, Washington, DC; Walter Reed Army Institute of Research, Silver Spring, Maryland; University of Michigan, Ann Arbor, Michigan; MedStar Washington Hospital Center, Washington, DC; MedStar Washington Hospital Center, Grand Prairie, Texas; Naval Medical Research Unit San Antonio, JBSA-Fort Sam Houston, San Antonio, Texas; Firefighters’ Burn and Surgical Research Laboratory, Washington, DC; MedStar Health, Georgetown University School of Medicine, Washington, DC; Walter Reed Army Institute of Research, Silver Spring, Maryland; University of Michigan, Ann Arbor, Michigan; MedStar Washington Hospital Center, Washington, DC; MedStar Washington Hospital Center, Grand Prairie, Texas; Naval Medical Research Unit San Antonio, JBSA-Fort Sam Houston, San Antonio, Texas; Firefighters’ Burn and Surgical Research Laboratory, Washington, DC; MedStar Health, Georgetown University School of Medicine, Washington, DC; Walter Reed Army Institute of Research, Silver Spring, Maryland; University of Michigan, Ann Arbor, Michigan; MedStar Washington Hospital Center, Washington, DC; MedStar Washington Hospital Center, Grand Prairie, Texas; Naval Medical Research Unit San Antonio, JBSA-Fort Sam Houston, San Antonio, Texas; Firefighters’ Burn and Surgical Research Laboratory, Washington, DC; MedStar Health, Georgetown University School of Medicine, Washington, DC; Walter Reed Army Institute of Research, Silver Spring, Maryland; University of Michigan, Ann Arbor, Michigan; MedStar Washington Hospital Center, Washington, DC; MedStar Washington Hospital Center, Grand Prairie, Texas; Naval Medical Research Unit San Antonio, JBSA-Fort Sam Houston, San Antonio, Texas; Firefighters’ Burn and Surgical Research Laboratory, Washington, DC; MedStar Health, Georgetown University School of Medicine, Washington, DC; Walter Reed Army Institute of Research, Silver Spring, Maryland; University of Michigan, Ann Arbor, Michigan; MedStar Washington Hospital Center, Washington, DC; MedStar Washington Hospital Center, Grand Prairie, Texas; Naval Medical Research Unit San Antonio, JBSA-Fort Sam Houston, San Antonio, Texas; Firefighters’ Burn and Surgical Research Laboratory, Washington, DC; MedStar Health, Georgetown University School of Medicine, Washington, DC; Walter Reed Army Institute of Research, Silver Spring, Maryland; University of Michigan, Ann Arbor, Michigan; MedStar Washington Hospital Center, Washington, DC; MedStar Washington Hospital Center, Grand Prairie, Texas; Naval Medical Research Unit San Antonio, JBSA-Fort Sam Houston, San Antonio, Texas; Firefighters’ Burn and Surgical Research Laboratory, Washington, DC; MedStar Health, Georgetown University School of Medicine, Washington, DC; Walter Reed Army Institute of Research, Silver Spring, Maryland; University of Michigan, Ann Arbor, Michigan; MedStar Washington Hospital Center, Washington, DC; MedStar Washington Hospital Center, Grand Prairie, Texas; Naval Medical Research Unit San Antonio, JBSA-Fort Sam Houston, San Antonio, Texas; Firefighters’ Burn and Surgical Research Laboratory, Washington, DC; MedStar Health, Georgetown University School of Medicine, Washington, DC; Walter Reed Army Institute of Research, Silver Spring, Maryland; University of Michigan, Ann Arbor, Michigan; MedStar Washington Hospital Center, Washington, DC; MedStar Washington Hospital Center, Grand Prairie, Texas; Naval Medical Research Unit San Antonio, JBSA-Fort Sam Houston, San Antonio, Texas; Firefighters’ Burn and Surgical Research Laboratory, Washington, DC; MedStar Health, Georgetown University School of Medicine, Washington, DC

## Abstract

**Introduction:**

Severe burn injury induces a profound systemic molecular response. Yet, the various molecular mechanisms mediating clinical outcomes after burn injury are not well characterized. Using longitudinal global gene expression data, we investigate the evolution of transcriptomic responses to thermal injury. We hypothesized that burn patients with differing clinical outcomes will show diverging transcriptomic alterations over time.

**Methods:**

This prospective observational study included patients from a regional burn center from 2013 to 2017. Patient demographics, burn injury characteristics, and blood samples for mRNA extraction and microarray whole genome gene expression profiling were collected at admission and set timepoints until 30 days. Patients were divided into 4 groups based on mortality status and hospital length of stay (LOS); G1 - died within 7 days, G2 - died after 7 days, G3 - discharged after 7 days, and G4 - discharged within 7 days. Transcriptomic abundances were compared between groups to identify differences in gene profiling. Pathway enrichment analysis was performed to determine the top significant pathways in each group.

**Results:**

A total of 116 patients were analyzed. Most patients were male (72.4%) with a median age of 39 and TBSA of 12.3%. Overall mortality rate was 12.9%. The distribution of patients in G1 to G4 was 8%, 5%, 60%, and 27% respectively. A total of 1,245 blood samples and 17,289 transcripts were quantified. At admission, genes were differentially expressed in G1, G2 and G3 compared to G4 (FDR-corrected P < 0.05 and magnitude of log fold-change > 1). Significant gene sets were largely nonoverlapping, with only a few immune response related genes being activated across all groups. Pathway enrichment analysis showed significant up-regulated protein folding pathways in G1, and significant up-regulated pathways related to oxidative stress and temperature homeostasis in G3. Intra-individual transcriptomic changes from admission to 30 days showed distinct group- and time-dependent patterns. G4 showed the least pronounced transcriptomic response without any noticeable change over time, while G3 mounted a transcriptomic response that increased over time.

**Conclusions:**

We identified transcriptomic alterations associated with distinct outcomes related to mortality and hospital LOS after burn injury. Alterations among groups were present at admission and persisted for 30 days. Connecting transcriptomic alterations to molecular mechanisms continues to evolve.

**Applicability of Research to Practice:**

Enhanced understanding of transcriptomic alterations and molecular mechanisms of burn injury trajectories will allow for the development of early biomarkers, prognostic tools and novel intervention strategies.

